# Experimental evaluation of a real-time implementation of compensatory reserve measurement in a human model of hemorrhagic shock

**DOI:** 10.3389/fbioe.2026.1756626

**Published:** 2026-04-15

**Authors:** Ryan Ortiz, Jose M. Gonzalez, Tina Rodgers, Sofia I. Hernandez Torres, Victor A. Convertino, Eric J. Snider

**Affiliations:** 1 Organ Support & Automation Technologies Department, U.S. Army Institute of Surgical Research, San Antonio, TX, United States; 2 Battlefield Health & Trauma Center for Human Integrative Physiology, U.S. Army Institute of Surgical Research, San Antonio, TX, United States; 3 Department of Medicine, Uniformed Services University of the Health Sciences, Bethesda, MD, United States; 4 Department of Emergency Medicine, University of Texas Health Science Center, San Antonio, TX, United States; 5 Department of Surgery, University of Texas Health Science Center, San Antonio, TX, United States

**Keywords:** hemorrhage, machine learning, medical devices, physiology, sensors, triage, wearable healthcare device

## Abstract

**Introduction:**

The leading cause of preventable traumatic death is hemorrhage. Early detection of hemorrhagic shock remains a critical challenge. For the early prediction of hemorrhagic shock-related cardiovascular decompensation, our team has developed the compensatory reserve measurement (CRM) algorithm. CRM uses a photoplethysmography waveform to quantify the body’s capacity to compensate during hypovolemia. This study focuses on the development and use of an application that can predict CRM in real-time (CRM_RT_) during simulated hypovolemia experiments.

**Methods:**

The CRM_RT_ application was developed in Python to generate CRM predictions and highlight trend trajectories in real-time (RT). Data were collected during a human research protocol that was reviewed and approved by the Institutional Review Board. Participants (n = 20) meeting the inclusion criteria underwent a simulated hypovolemia procedure in a lower-body negative pressure chamber while wearing a Masimo® MightySat® Rx pulse oximeter. Data were streamed in RT via a Bluetooth® connection to a computer running the CRM_RT_ application.

**Results:**

CRM was successfully implemented for RT data capture during the research study. The CRM_RT_ application achieved a median performance error of −0.95%, while the median absolute performance error was higher at 19.00%. CRM_RT_ resulted in an average early prediction time of 18.3 min by tracking the slope trend changes in RT.

**Discussion:**

The CRM_RT_ application effectively tracked CRM during simulated hypovolemia using a wearable non-invasive sensor. Predictions served as an earlier indicator of hemorrhage compared to traditional vital signs, addressing a limitation of current triage practices. Overall, the CRM_RT_ application represents a promising advancement toward RT prediction of hypovolemic decompensation.

## 1 Introduction

Hemorrhage is one of the leading causes of preventable death following trauma in both civilian and military populations. Most trauma fatalities occur early, often within hours to minutes, with uncontrolled bleeding as the predominant cause (Bellamy, 1984). Combat casualty data from the operations in Iraq and Afghanistan reinforced this observation, identifying hemorrhage as the primary cause of potentially survivable battlefield deaths (Holcomb et al., 2007; Eastridge et al., 2012). Despite advancements in trauma systems and prehospital care, early detection of hemorrhagic shock remains a persistent clinical challenge (Bonanno, 2020). During the initial compensated phase of blood loss, the body activates a cascade of physiological mechanisms such as vasoconstriction, tachycardia, and fluid redistribution to maintain adequate perfusion to vital organs (Muir, 2006). These compensatory responses can mask the severity of hemorrhage, rendering traditional vital signs such as heart rate, systolic blood pressure, and mental status unreliable indicators of early shock (Holcomb et al., 2007; Sauaia et al., 2020). Consequently, patients are often under-triaged or treated too late, leading to hypoxia (Fecher et al., 2021), metabolic acidosis (Klepper et al., 1988), multi-organ failure (Tremoleda et al., 2017), or death. Timely recognition of hemorrhagic shock before overt cardiovascular decompensation is essential for guiding resuscitation, initiating transfusion protocols, and improving survival.

To overcome the limitations of traditional vital signs and improve early detection, the U.S. Army Institute of Surgical Research (USAISR) has developed the compensatory reserve measurement (CRM), an artificial intelligence algorithm that estimates the level of physiological compensation experiencing hypovolemia (Convertino et al., 2020). The CRM algorithm was developed using deep learning methodologies, particularly a convolutional neural network (CNN)-based architecture that can extract features from high-fidelity biological waveforms (Techentin, 2019). CRM can quantify the body’s remaining capacity to compensate during hypovolemia by analyzing continuous arterial waveform (Moulton et al., 2013). The output of the algorithm provides a value on a scale of 0%–100%, where 100% indicates full compensatory reserve status and 0% indicates the threshold of decompensated shock. Unlike static thresholds, CRM provides a dynamic and individualized measure of the physiological status, capturing nonlinear patterns of decompensation that vary between individuals (Convertino et al., 2016; Bedolla et al., 2023). This enables earlier recognition of cardiovascular deterioration and provides a crucial window for intervention during the compensated phase (Convertino et al., 2022).

The development of the CRM algorithm required high-fidelity physiological waveform data during hemorrhage up to the point of hemodynamic decompensation, i.e., the complete exhaustion of the body’s ability to compensate to maintain blood pressure, resulting in a sudden decrease in blood pressure (Shen and Baker, 2015). A lower-body negative pressure (LBNP) experimental protocol was performed to capture hemorrhagic data to the point of hemodynamic decompensation. The LBNP procedure induces a simulated hemorrhage by placing the lower section of a participant’s body into a negative-pressure chamber that is sealed around the waist. By applying progressively increasing levels of negative pressure, the participant’s blood volume is redistributed to the lower half of their body, causing central hypovolemia (Goswami et al., 2019). This provided a reproducible and ethical experimental model to simulate hemorrhage for capturing data to detect changes associated with compensatory failure and develop the CRM algorithm, along with testing and validating CRM in a controlled laboratory setting (Convertino et al., 2016).

The translation of CRM from the laboratory setting to clinical practice has been achieved through advances in signal processing methodologies and improved wearable technologies (Matthews et al., 2023). Recent validation studies have demonstrated that CRM values can be accurately predicted from photoplethysmography (PPG) waveforms, enabling real-time (RT) monitoring of CRM values (CRM_RT_) using non-invasive and wearable clinical sensors (Roden et al., 2024). The integration of wearable technologies with CRM can extend its potential beyond hospital settings to prehospital, austere, and resource-constrained environments where clinical monitoring is limited (Di Carlo et al., 2021), (Bixio et al., 2024). Additionally, CRM_RT_ will enable rapid assessment of potential hemorrhage trauma in prolonged field care (PFC) scenarios, where rapid and continuous monitoring of physiological compensatory reserve mechanisms can limit potentially preventable deaths (Schlotman et al., 2019).

To move toward PFC testing, field validation of CRM has been completed to demonstrate its usability in the field and the clinical impact in operationally relevant scenarios. During the 2024 Army Expeditionary Warrior Experiment, more than 90 soldiers—including both medics and non-medical personnel—evaluated CRM using an intuitive tablet interface that displayed values as a color-coded fuel gauge (green >70%, yellow 40%–69%, and red <40%) zones alongside traditional vital signs (Convertino et al., 2025). Participants correctly identified physiological deterioration in 93% of simulated casualties, with CRM detecting impending decompensation more than 15 min before the occurrence of cardiovascular decompensation. In a subsequent urban battlefield scenario involving mass casualty conditions, participants successfully integrated CRM into triage and evacuation decision-making, demonstrating its feasibility as a field-ready tool for PFC.

To further develop the CRM algorithm, the integration of the algorithm with an FDA-cleared, widely available portable clinical monitoring device would broaden its impact and availability in clinical settings. The present study addresses this translation by implementing CRM_RT_ using a Masimo® MightySat® Rx pulse oximeter (Masimo Corporation, Irvine, California, United States of America), an FDA-cleared clinical monitoring platform available in both clinical and prehospital settings. The real time implementation of the portable technology was validated using LBNP to compare it against traditional sensor monitors and evaluate the early prediction capability for CRM_RT_. This work represents a critical step toward the advancement of CRM from an experimental tool to an operationally deployable solution for improving early hemorrhage detection, triage accuracy, and survival outcomes in both civilian and military trauma systems.

## 2 Methods

To describe the development and evaluation of CRM_RT_, this section first demonstrates how the CRM_RT_ application functions with an overview of the application user interface, followed by a description of how data were captured in human subjects using LBNP to simulate hypovolemia. These data were then used to evaluate the CRM_RT_ application and compared against retrospectively processed CRM calculations from different PPG inputs. Statistical analysis for this comparison and the evaluation of CRM_RT_ for its capacity for early hemorrhage detection are described in the final methodological section.

### 2.1 Real-time compensatory reserve measurement application

#### 2.1.1 Algorithm implementation

The CRM_RT_ application utilizes a previously developed one-dimensional CNN model trained on arterial waveforms collected from retrospective human LBNP studies (Techentin, 2019; Convertino et al., 2022). The CRM algorithm quantifies the percentage of the remaining physiological compensatory capacity, with a 100% value indicating full reserve and 0% signifying imminent cardiovascular decompensation.

The algorithm requires PPG waveform data sampled at 100 Hz. Since the MightySat® Rx pulse oximeter samples at a different native frequency, signal resampling was performed using the resample function from the SciPy Python library (version 1.15.3) (Virtanen et al., 2020), which implements the Fourier method for frequency conversion. The CNN model operates on fixed-length PPG waveform segments corresponding to a 5-s window (500 samples at 100 Hz). In the CRM_RT_ application, these windows are processed causally in real-time, using only the current and preceding samples without access to future data. Additionally, the waveform amplitude was normalized to a 0–1 scale using min–max normalization (Equation 1):

$$x^{\prime }=\frac{\;x_{i\;}-\min\left(x\right)}{\max\left(x\right)-\min\left(x\right)}.$$ (1)

Here, x_i_ represents each sample point in the PPG waveform segment, and min(x) and max(x) are the minimum and maximum amplitude values, respectively, within that segment.

The CRM_RT_ application uses the CNN model to generate CRM predictions at 1 Hz, which is computed using a 5-s sliding window with a 1-s stride. To reduce beat-to-beat variability in the output, a 20-s trailing moving average was applied to the raw CRM predictions. This smoothing was implemented using a causal rolling mean (window = 20 samples and minimum periods = 20), ensuring that only the current and past CRM values contributed to each smoothed estimate, which is consistent with RT operational constraints.

#### 2.1.2 Hardware and data transmission

The MightySat® Rx pulse oximeter transmits PPG waveforms and vital-sign signal data via Bluetooth low energy (BLE) to CRM_RT_ running on a Windows-based device. The application was designed to connect with a single MightySat® Rx device and process incoming data through parallel pipelines for real-time visualization and CRM calculation.

#### 2.1.3 User interface design

**FIGURE 1:**
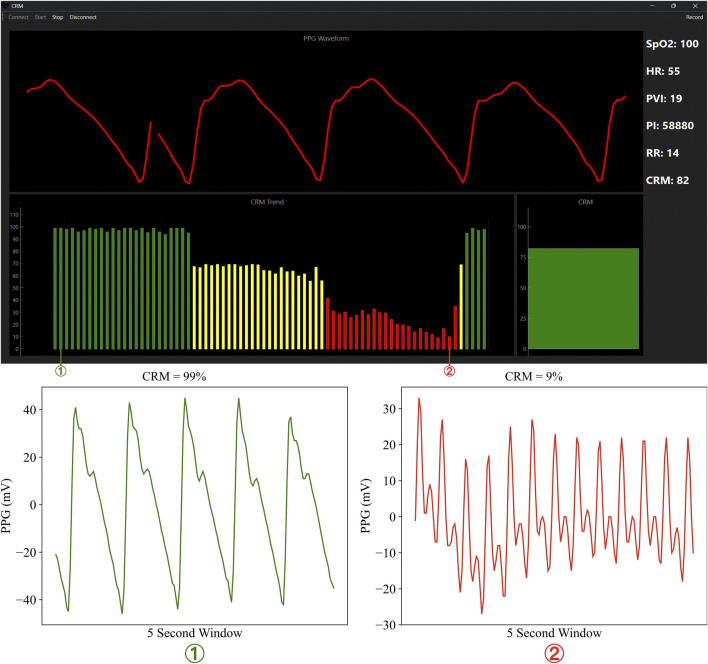
Overview of the CRM_RT_ application. (Top) Graphical user interface showing real-time waveform and derived vital signs. The bottom section of the interface highlights CRM predictions in real-time and the overall trends for the patient. (Bottom) It highlights how the PPG waveform differs for the high (green zone, labeled ①) and low (red zone labeled ②) CRM values.

The CRM_RT_ application user interface was designed following the principles of providing the user(s) with essential monitoring information without causing a cognitive burden when using the application. The layout incorporates visual elements that are familiar to healthcare providers as it aims to share similarities with standard patient monitors to facilitate a smoother transition for the operator. Four primary display components provide comprehensive patient monitoring (Figure 1):

- Real-time PPG waveform display for signal quality assessment.
- Vital-sign panel showing CRM, oxygen saturation, heart rate, respiratory rate, and other vitals provided by the MightySat® Rx pulse oximeter.
- Instantaneous CRM value displayed as a visual “fuel gauge” indicator.
- Trend graph of CRM over the monitoring period.

The trend graph plays a key role in the triage of a patient’s status as it provides a contextualized aspect to CRM monitoring. For example, a momentarily low CRM value accompanied by a stable or improving trend may indicate to an operator that this patient is less urgent than someone who has a similar low CRM but is accompanied by a decreasing CRM trend.

### 2.2 Lower-body negative pressure human research study

#### 2.2.1 Ethical approval and study population

This research was conducted in compliance with a protocol that was reviewed and approved by the Headquarters U.S. Army Medical Research and Development Command Institutional Review Board. The study included healthy participants who met the following inclusion criteria: (1) normotensive (<140/90) male or female individuals, (2) between 18–65 years old, (3) who were military or civilians, (4) had a waist circumference between 22–42 inches, and (5) were willing to refrain from exercise and stimulants for the 24 h prior to the study (including caffeine, alcohol, and herbal medications). All female volunteers of childbearing potential were also required to have a documented negative pregnancy test within 24 h prior to the study. Individuals who met these criteria were then evaluated against the exclusion criteria and the contraindications listed in Supplementary Methods. Participation in the study was completely voluntary, and participants were compensated for their participation. After participants were confirmed eligible based on the inclusion and exclusion criteria, consent was obtained from the volunteers either prior to their study day or on the same day. For the LBNP study, the clinician administered a health questionnaire, followed by the completion of a screening form to ensure that all criteria were met.

#### 2.2.2 Experimental setup

Once the screening procedure was satisfactory, the participant was fitted with a kayak skirt and secured to the LBNP chamber in the supine position, with their lower body, from the iliac crest downward, sealed within the LBNP chamber. For this analysis, relevant data were collected from two PPG sensors, namely, (1) a Masimo LNCS-DCI pulse oximeter clip connected to a patient monitor (Delta XL, Dräger, Lübeck, Germany) and (2) a MightySat® Rx pulse oximeter connected wirelessly to the CRM_RT_ application. The patient monitor provided high-fidelity PPG waveform capture at 1,000 Hz via a PowerLab Data Acquisition System (ADInstruments, Sydney, Australia) and LabChart software. The CRM_RT_ application was able to record the raw PPG waveform in CSV format at 31.25 Hz. For the analysis of the CRM_RT_, the first 20 datasets collected in the study were used as three participants withdrew after providing consent but prior to any data collection.

#### 2.2.3 LBNP protocol

After all sensors were placed on the participant and data recording began, a stabilization period of 5-min was used to record baseline data. Then, negative pressure was applied in stepwise progressive increments reaching −15, −30, −45, −60, −70, −80, −90, and −100 mmHg over 5-min stages. The procedure would be stopped at any point at the request of the participant or the medical monitor, and if any pre-syncopal symptoms were apparent in the participant’s vital signs, such as systolic blood pressure under 80 mmHg, a significant decrease in heart rate, SpO_2_ below 90%, or a sudden change in any vital sign as determined by the medical monitor, or if the participant expressed any symptoms related to hemodynamic decompensation. Upon reaching the endpoint criteria, negative pressure was immediately released, and the participant was monitored through a 10-min recovery period to ensure the return of vital signals and symptoms to baseline status. Once the recovery period ended, data recording was stopped, and all sensors were removed from the participant.

### 2.3 Retrospective compensatory reserve data processing

The PPG waveforms of both the MightySat® Rx and Dräger Infinity were retrospectively processed through the CRM algorithm. The high-fidelity Dräger waveforms, originally sampled at 1,000 Hz, were reduced to 100 Hz by taking every 10th sample to match the algorithm’s input frequency. These waveforms were then normalized using the same min–max scaling applied in real-time. Additionally, the raw PPG waveforms from the MightySat® Rx were retrospectively processed using identical preprocessing and normalization procedures implemented in the CRM_RT_ application. These retrospective assessments enabled the comparison of different CRM performances against the ground-truth CRM and device-specific CRM estimation between clinical-grade and portable pulse oximetry.

In contrast to CRM_RT_, retrospective CRM processing was performed offline with access to the complete recorded PPG waveform for each subject. Although identical preprocessing, normalization, and model inference steps were applied, the absence of real-time constraints enabled consistent window alignment across the full recording. Therefore, CRM_RT_ and retrospective CRM are not expected to be numerically identical even when derived from the same subjects and sensors.

### 2.4 Statistical analysis

Analysis of the CRM_RT_ data-stream was based on two primary characteristics, namely, (i) similarity to CRM measured from other data-streams and (ii) assessment of early decompensation prediction. Starting with CRM data-stream comparisons, CRM_RT_ was compared with the calculated CRM ground-truth (CRM_GT,_ Equation 2), retrospective Dräger Infinity-based CRM (CRM  ∞ ), and retrospective MightySat® Rx-based CRM (CRM_MS_). To assess the prediction error with and without directionality, the median (MDPE) and absolute median (MDAPE) of performance errors (PEs) (Equation 3) were calculated for each data-stream vs. CRM_GT_. PE was defined as the relative difference between the ground-truth and predicted CRM, presented as a percentage error in which positive values indicate over-prediction and negative values indicate under-prediction. PE values were discarded when CRM_GT_ equaled 0. In addition, coefficients of variation (CV) for a 30-s moving window were measured across each dataset, and the median value for each data-stream were calculated for each participant to quantify localized noise in CRM signals.

CRMGT=1−LBNPtLBNPHDD (2)

PE=CRMPredicted−CRMGTCRMGT (3)

Confusion matrices were created for CRM_RT_ predictions compared to CRM_GT_ based on CRM color categories used in the user interface (green, yellow, and red; Figure 1). Binary performance metrics (accuracy, precision, recall, specificity) were calculated for each category to evaluate agreement between CRM_RT_ and CRM_GT_ classifications.

Each subject was analyzed through this methodology, and the overall results were summarized across the dataset. For the early detection of decompensation, three methodologies were derived from CRM_RT_ predictions. The first two were based on absolute CRM scores corresponding to color changes in the CRM_RT_ application, where “green” predictions become “yellow” or “red” indicators at 70% and 40%, respectively. A total of 1 Hz CRM predictions were assessed for passing the “yellow” or “red” threshold gate for at least 45 predictions in a 1-minute or 60-s prediction moving window. The third methodology was based on the slope trends of CRM predictions, where a significant trend was defined as a slope decrease of 15% CRM over a moving 5-min window; this threshold was set based on the initial review of a subset of data to identify consistent slope trends. This same trend methodology was applied to heart rate (HR) and MAP trends to see how these typical metrics could be used for tracking decompensation. Times when each of these methods initially flagged were logged for each methodology and compiled across the dataset. The results were compared to the times when the LBNP chamber was in use to confirm the logic flagged during the experimental run vs. initially or after the pressure chamber was released. If no flag for a method was indicated by a methodology, this result was noted, and a 0-min early indication was added for that participant.

For each analysis, datasets were assessed for normal distributions using the Shapiro–Wilk test, where *p*-values less than 0.05 indicated non-normal distributions. For non-parametric analyses (MDPE and MDAPE), the Friedman test and *post hoc* Dunn’s test were used for non-parametric data assessment. For the parametric analyses (detection times and CRM CV), repeated measures one-way ANOVA was used, along with *post hoc* Tukey’s test. *p*-values less than 0.05 indicate statistically significant differences and are denoted in the results when present.

## 3 Results

**FIGURE 2:**
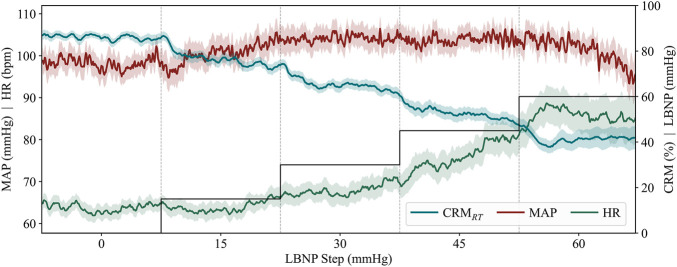
Average CRM_RT_ predictions, MAP, and HR during incremental LBNP pressure steps for all the analyzed subjects who reached at least 60 mmHg (n = 20 subjects). LBNP pressure profile (black line) is shown for experimental context. Error bars represent the standard deviation and are shown as shaded regions for CRM_RT_, MAP, and HR. Outlier data for one subject was removed and replaced with interpolated MAP predictions during baseline (0 mmHg LBNP step) due to error with MAP capture.

Reduction in the central blood volume due to LBNP has a delayed effect on arterial blood pressure, with the mean arterial pressure remaining steady or increasing slightly for multiple LBNP pressure steps (Figure 2). While MAP begins to decrease prior to the end of the LBNP experiment, CRM_RT_ continuously decreases throughout, providing an earlier indication of blood-loss effects.

**FIGURE 3:**
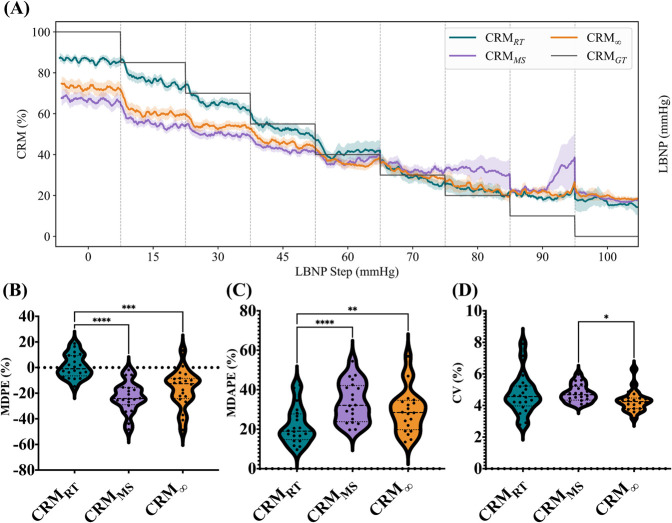
**(A)** CRM over-normalized LBNP steps across various PPG sources compared against the CRM_GT_, **(B)** MDPE, **(C)** MDAPE, and **(D)** CV across all CRM predictions. Asterisks denote statistically significant differences between groups when appropriate (*p < 0.05; **p < 0.01; ***p < 0.001; ****p < 0.0001).

In quantitatively evaluating CRM performance, we first compared it against two other CRM prediction approaches to confirm the accuracy of the MightySat® Rx device and the RT prediction methodology. The overall trends remained similar between the three approaches (Figure 3A). MDPE values for each vs. CRM_GT_ was −0.95% vs. −24.20% vs. −12.57% for CRM_RT_, CRM_MS_, and CRM  ∞ , respectively (Figure 3B). The CRM_RT_ MDPE was significantly lower than that of the two other methods. For MDAPE, CRM_RT_ was significantly lower than that of the two other CRM predictions at 19.00% vs. 32.07% (CRM_MS_) and 28.46% (CRM  ∞ , Figure 3C). While the RT predictions worked better than retrospective analysis, another important data trend is the overall variability of the prediction. Based on CRM CV for each method, the differences were not significant with CRM_RT_ at 4.71% vs. CRM_MS_ at 4.77% and CRM  ∞ at 4.29% (Figure 3D).

**FIGURE 4:**
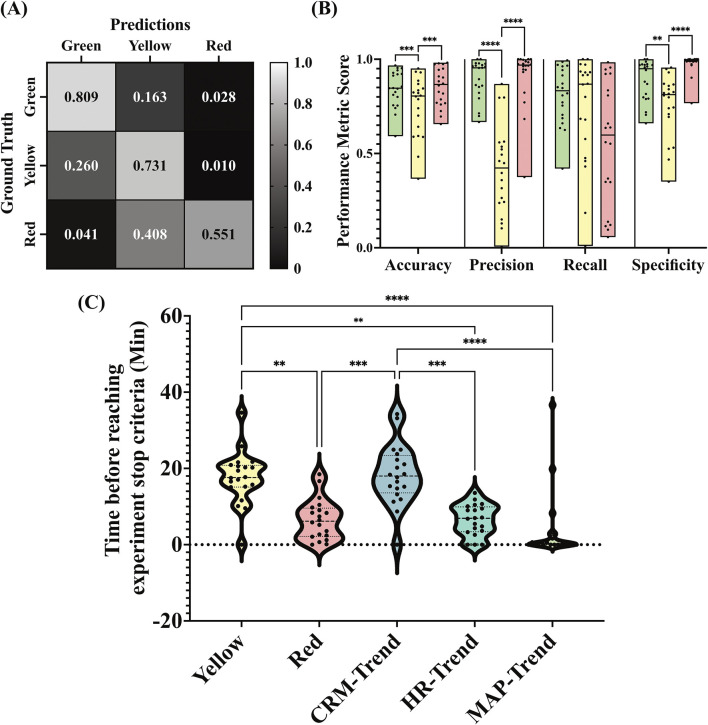
**(A)** Confusion matrix of CRM_RT_ predictions compared to CRM_GT_ for the “green,” “yellow,” and “red” categories. Results are shown as the average across all subjects (n = 20) and are normalized so that each GT category sums to 1 for each subject. **(B)** Accuracy, precision, recall, and specificity calculated for each GT category across all subjects (n = 20). **(C)** Time before reaching the experiment stop criteria for overall trends, yellow indicator, and red indicator (n = 20). Trends are calculated based on CRM, MAP, and HR. Asterisks denote statistically significant differences between GT categories when appropriate (**p < 0.01; ***p < 0.001; ****p < 0.0001).

To further assess the CRM_RT_ performance, we evaluated categorical predictions based on the “green”, “yellow”, and “red” zones defined in the user application (Figure 1). Based on these categorical predictions, the confusion matrix results indicate that the “green” and “yellow” categories were more often correctly predicted by CRM_RT_, while “red” predictions were falsely classified in the “yellow” zone (Figure 4A). Conventional performance metrics were measured based on binary class divisions for the three categories, with the results showing that the “green” and “red” categories have significantly stronger accuracy, precision, and specificity than the “yellow” category predictions (Figure 4B). The median sensitivity (recall) was 83%, 87%, and 60% for the green, yellow, and red zones, respectively. The median specificity was 95%, 81%, and 99% for the green, yellow, and red zones, respectively. Confusion matrix results comparing early prediction capability derived from CRM_RT_, conventional threshold approaches provided a 6.55 ± 5.27 min indication when waiting for the “red” zone to consistently trigger. Furthermore, only one (5%) subject was not predicted early with this approach. Using the “yellow” zone instead, 17.59 ± 6.92 min was the average time for early prediction. Similarly, one case was not predicted with this approach as it started the experiment in the yellow CRM prediction range. The third methodology was based on the trend decrease in CRM from the baseline. Using this methodology, the early prediction window was 18.30 ± 7.94 min, with one subject not triggering the prediction. This same trend methodology was quantified using MAP and HR instead of CRM, with each method performing worse than CRM at 3.71 ± 9.03 min and 6.45 ± 4.08 min, respectively. Both the trend and yellow indicator approaches were significantly quicker than the red indicator approach, but they were not significantly different from each other (Figure 4C). Overall, the CRM_RT_ approach was successful in the early prediction of hemodynamic decompensation, which is a critical step in the deployment of the CRM triage tool.

## 4 Discussion

Hemorrhagic shock is one of the leading causes of potentially preventable trauma deaths in both civilian and military prehospital settings, underscoring the critical importance of early recognition and intervention (Bellamy, 1984; Eastridge et al., 2012; Sauaia et al., 2020). Despite continued advancement of trauma care capabilities, the fundamental challenge of early and timely identification and treatment of hemorrhagic shock still remains. Timely intervention is essential as approximately 99% of trauma patients experiencing hypovolemic shock of classes I or II survive, while survival decreases as class IV shock is reached, with mortality exceeding 30% (Parks et al., 2020). This narrow window for effective intervention demonstrates the need for detection capabilities that can identify hemorrhage during the earliest stages of hypovolemia.

However, traditional vital signs are typically masked by the body’s compensatory mechanisms in the early stages of hemorrhagic shock, hiding the underlying hemorrhagic trauma. Although the ideal intervention time in hypovolemic shock is in classes I or II, this is complicated as casualties often maintain seemingly normal traditional vital signs that remain stable or show only subtle changes during these early classes of hypovolemic shock until their physiological compensation reserve is exhausted, corresponding to hypovolemic shock classes III and IV, at which point life-saving interventions may no longer be effective (Kuo and Palmer, 2022). This creates a critical gap where, ideally, intervention should occur in hypovolemic classes I and II, but conventional monitoring fails to identify hemorrhagic shock during this phase.

A predictive method capable of identifying cardiovascular decompensation before traditional vital signs appear abnormal is essential for improving trauma outcomes. Previous work has demonstrated that LBNP correlates with blood loss that encompasses hypovolemic shock classes I and II blood-loss definitions (Hinojosa-Laborde et al., 2014), providing a controlled experimental model for evaluating early prediction capabilities. This study utilized the LBNP procedure to assess CRM’s ability to provide an early indication of impending cardiovascular decompensation and whether this predictive capability translates from retrospective analysis to real-time implementation. CRM_RT_ had the lowest MDPE (−0.95%), but its MDAPE (19.00%) was notably higher, indicating that there was error, although it was symmetrical for CRM_GT_. Strong performance scores were evident from categorical zone predictions using CRM_RT_, with accuracies for each category prediction all exceeding 80%. Conversely, CRM_MS_ and CRM  ∞ showed higher error for both MDPE and MDAPE; therefore, overall, CRM_RT_ appeared to perform better. The initial concern with RT-based predictions was the introduction of additional errors, but the opposite was shown to be true. The exact reason for this is unknown; however, future work will assess CRM  ∞ in RT to evaluate the impact on the results and different pre-processing methods of the data to understand the performance differences. Overall, CRM_RT_ performed strongly, indicating its utility for this triage application.

As demonstrated in Figure 2, MAP remained relatively stable in the first few steps of the LBNP protocol, while CRM_RT_ progressively declined throughout the study from the baseline value. CRM_RT_ enabled early prediction of decompensation 18.30 ± 7.94 min based on trend-based detection, which was comparable to the yellow color indicator approach at 15.64 ± 10.01. While each approach was similar, there was a higher failure rate with the color threshold (4 out of 20 participants), caused by the participant’s CRM value starting below the 0.75 threshold. Only one participant failed to trigger the prediction with the trend approach, which was equivalent to the failure rate with the red color threshold. However, this approach only provided a 6.55 ± 5.27 min early indication. These results strongly indicate that the early trend detection approach is most appropriate, with the earliest detection capability and lowest failure rate. This approach synergizes well with the CRM_RT_ user application, which plots data trends in RT. These early indication times are clinically important as they expand the intervention window during which the participants retain physiological reserve and are most likely to respond to resuscitative efforts in an actual trauma casualty.

The 18.30-min average early prediction time demonstrated by CRM_RT_ has significant implications for clinical intervention. This warning window enables time-critical actions that would not be possible otherwise when relying on traditional vital signs alone (Muniz et al., 2013). In prehospital and military settings, early identification of impending decompensation can facilitate expedited triage and transport decisions (Lokerman et al., 2025), earlier initiation of damage control resuscitation and blood product administration (Cap et al., 2018), application of hemorrhage control measures such as tourniquets or wound packing (Albeshri, 2025), and activation of trauma response teams prior to patient arrival (Franklin et al., 2000). In austere environments where resources are limited, this predictive capability may inform critical allocation decisions when multiple casualties arrive at the same time (Martin and Eckert, 2016). By identifying casualties in hypovolemic shock classes I and II before compensatory mechanisms are exhausted (Convertino et al., 2016), CRM_RT_ aims to address the gap between when intervention is most effective and when traditional monitoring detects hemodynamic abnormalities.

It is important to note that this study has several limitations that should be discussed along with future work to potentially address them. First, the evaluation of the CRM algorithm made use of LBNP as an alternative to hemorrhagic shock in screened healthy volunteers under controlled laboratory conditions. Consequently, CRM_GT_ is derived from LBNP protocol parameters rather than a direct physiological measurement of CRM; in the absence of a gold-standard measurement, this formulation serves as the accepted reference within this protocol. Casualties in the prehospital trauma care may present heterogeneous baseline values (Chen et al., 2009). These heterogeneous baseline values may be affected by injury type/severity, comorbidities (Tan et al., 2004), or even cardiovascular-targeting medications/age (Kim, 2021). In addition, the PPG signal in the field may be deteriorated by severe motion artifacts (Maeda et al., 2011). To address these issues, it is imperative to evaluate and collect data on the CRM algorithm in different settings, including, but not limited to, simulated field exercises and emergency trauma situations.

There are additional limitations related to the CRM_RT_ application; for example, it can only monitor a single patient, which limits its utility in mass casualty scenarios where rapid triage of multiple casualties is essential. In addition, the current CRM_RT_ application is limited to the MightSat® Rx pulse oximeter platform. Pairing with additional monitors or more wearable formats can allow for an even wider use of this technology. Future development will be carried out to allow multi-patient monitoring capabilities that enable providers to continuously assess CRM across multiple casualties simultaneously. This would enable the identification of casualties approaching decompensation even when they remain stable according to traditional vital monitoring, providing a standard metric for the prioritization of evacuation and resource allocation. In addition, due to the rapid advancement of wearable health technologies, particularly continuous physiological monitoring devices, there is a need to expand CRM deployment to take advantage of these technological advancements. Integration into wearable sensing devices would allow PFC monitoring, continuous assessment of soldier status during combat operations, and detection of hemorrhage prior to traditional vital-sign symptoms. This will require the validation of CRM accuracy across diverse PPG sensors to enable widespread adoption in wearable sensors.

## Data Availability

The data presented in this study are not publicly available because they have been collected and maintained in a government-controlled database located at the U.S. Army Institute of Surgical Research. This data can be made available through the development of a Cooperative Research and Development Agreement (CRADA) with the corresponding author.
