# Supplementary material for: Experimental evaluation of a real-time implementation of compensatory reserve measurement in a human model of hemorrhagic shock
Source: Front Bioeng Biotechnol. 2026 Apr 15;14:1756626. doi: 10.3389/fbioe.2026.1756626 (PMC13125002; doi:10.3389/fbioe.2026.1756626)
Supplement: Supplementary file 1 [file Supplementaryfile1.docx]

Supplementary Material

# Supplementary Methods

The following lists were used to assess participants who volunteered to participate in the study.

LNBP Inclusion criteria:

- Healthy, normotensive (<140/90) males or females
- Age 18 to 65 years
- Military or civilian from the general population
- Documentation of a negative pregnancy test within 24 hours prior to each study period if necessary-protocol specific: all women with child-bearing potential will complete a urine pregnancy test before the start of the study
- Waist circumference measurement required to be between a minimum of 22 inches and max of 42 inches
- Willing to refrain from exercise and stimulants such as caffeine, alcohol, and herbal medications 24 hours prior to their procedure in the LBNP chamber and verbally confirmed by volunteer

LBNP Exclusion criteria:

- Individuals with a history of alcohol or drug abuse
- Individuals who are pregnant, trying to become pregnant, or breastfeeding
- Individuals who have used any form of nicotine, regardless of whether it is in the form of smoking, smokeless tobacco, e-cigarettes, vaping or medication within the last 6 months
- Individuals with Type 1 or Type 2 diabetes
- Individuals with known or suspected abdominal hernias
- Individuals with a history of anaphylaxis
- Individuals with a history or family history of abnormal blood clotting, clots in deep veins in the legs or pelvis, or blood clots to the lungs
- Individuals with a history of pre-syncopal/syncopal (fainting) episodes
- Individuals with a history of orthostatic hypotension (low blood pressure that occurs from moving to a standing position from sitting or lying down)
- Individuals taking antihypertensive (blood pressure) medications
- Individuals with a history of or current respiratory illness(es) (e.g., Asthma, Chronic Obstructive Pulmonary Disease, Reactive Airway Disease, etc.)
- Individuals with autonomic dysfunction such as Shy-Drager Syndrome. Bradbury-Eggleston syndrome, sinus arrhythmia, idiopathic orthostatic hypotension, fainting disorders, etc.
- Individuals currently taking medications (e.g., cholinomimetics/cholinesterase antagonists, anticholinergics, adrenoreceptor agonists/sympathomimetics, and adrenoreceptor antagonists) that are known to alter the specific part of the nervous system that controls normal cardiovascular functions (e.g., the autonomic nervous system).
- Individuals with renal insufficiency or renal failure
- Individuals with varicose veins
- Individuals with gynecoid or central obesity
- BMI.39 kg/m^2^
- Participants with silicone allergies
- Participants with implanted medical devices unless cleared by clinician (i.e., arm implanted birth control devices)

LBNP Contraindications:

- Are having surgical procedures
- Anticipated or known history of difficult airway
- Have severe claustrophobia and/or anxiety in close or confined spaces
- Have anatomical abnormalities that would interfere with clinical care
- Have communication disorders that would interfere with clinical care
- Have uncontrolled movements that would prevent the participant from remaining enclosed in the chamber
- Have known or suspected inguinal hernias
